# Multicenter Evaluation of a Weakly Supervised Deep Learning Model for Lymph Node Diagnosis in Rectal Cancer at MRI

**DOI:** 10.1148/ryai.230152

**Published:** 2024-02-14

**Authors:** Wei Xia, Dandan Li, Wenguang He, Perry J. Pickhardt, Junming Jian, Rui Zhang, Junjie Zhang, Ruirui Song, Tong Tong, Xiaotang Yang, Xin Gao, Yanfen Cui

**Affiliations:** From the Department of Medical Imaging, Suzhou Institute of Biomedical Engineering and Technology, Chinese Academy of Sciences, Suzhou, China (W.X., J.J., R.Z., X.G.); Department of Radiology, Shanxi Province Cancer Hospital/Shanxi Hospital Affiliated to Cancer Hospital, Chinese Academy of Medical Sciences/Cancer Hospital Affiliated to Shanxi Medical University, Taiyuan 030013, China (D.L., J.Z., R.S., X.Y., X.G., Y.C.); Department of Radiology, the First Affiliated Hospital, Zhejiang University School of Medicine, Hangzhou, China (W.H.); Department of Radiology, University of Wisconsin School of Medicine and Public Health, E3/311 Clinical Science Center, Madison, Wis (P.J.P.); Department of Radiology, Fudan University Shanghai Cancer Center, Shanghai, China (T.T.); Department of Oncology, Shanghai Medical College, Fudan University, Shanghai, China (T.T.); and Guangdong Provincial Key Laboratory of Artificial Intelligence in Medical Image Analysis and Application, Guangzhou, China (Y.C.).

**Keywords:** MR Imaging, Abdomen/GI, Rectum, Computer Applications-Detection/Diagnosis

## Abstract

**Purpose:**

To develop a Weakly supervISed model DevelOpment fraMework (WISDOM) model to construct a lymph node (LN) diagnosis model for patients with rectal cancer (RC) that uses preoperative MRI data coupled with postoperative patient-level pathologic information.

**Materials and Methods:**

In this retrospective study, the WISDOM model was built using MRI (T2-weighted and diffusion-weighted imaging) and patient-level pathologic information (the number of postoperatively confirmed metastatic LNs and resected LNs) based on the data of patients with RC between January 2016 and November 2017. The incremental value of the model in assisting radiologists was investigated. The performances in binary and ternary N staging were evaluated using area under the receiver operating characteristic curve (AUC) and the concordance index (C index), respectively.

**Results:**

A total of 1014 patients (median age, 62 years; IQR, 54–68 years; 590 male) were analyzed, including the training cohort (*n* = 589) and internal test cohort (*n* = 146) from center 1 and two external test cohorts (cohort 1: 117; cohort 2: 162) from centers 2 and 3. The WISDOM model yielded an overall AUC of 0.81 and C index of 0.765, significantly outperforming junior radiologists (AUC = 0.69, *P* < .001; C index = 0.689, *P* < .001) and performing comparably with senior radiologists (AUC = 0.79, *P* = .21; C index = 0.788, *P* = .22). Moreover, the model significantly improved the performance of junior radiologists (AUC = 0.80, *P* < .001; C index = 0.798, *P* < .001) and senior radiologists (AUC = 0.88, *P* < .001; C index = 0.869, *P* < .001).

**Conclusion:**

This study demonstrates the potential of WISDOM as a useful LN diagnosis method using routine rectal MRI data. The improved radiologist performance observed with model assistance highlights the potential clinical utility of WISDOM in practice.

**Keywords:** MR Imaging, Abdomen/GI, Rectum, Computer Applications-Detection/Diagnosis

*Supplemental material is available for this article*.

Published under a CC BY 4.0 license.

## Graphical abstract

**Figure:**
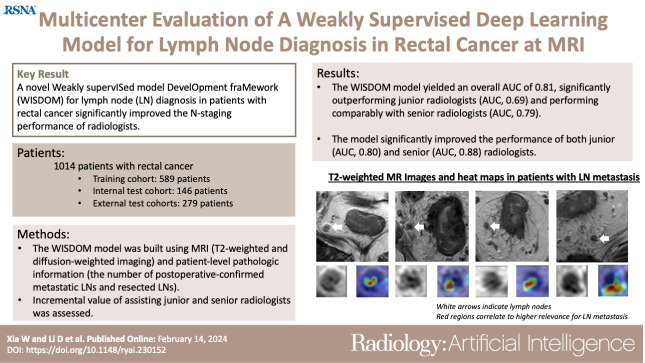

**Summary**

A weakly supervised deep learning–based lymph node diagnosis model for rectal cancer using preoperative MRI data coupled with postoperative patient-level pathologic information significantly improved the N staging performance of radiologists.

**Key Points**

■ An emerging Weakly supervISed model DevelOpment fraMework (WISDOM) model for lymph node diagnosis in patients with rectal cancer was developed and tested on three cohorts, yielding favorable N staging performance (overall area under the receiver operating characteristic curve [AUC] = 0.81).
■ Radiologists had improved N staging performance with the assistance of the WISDOM model (junior radiologists: AUC = 0.69 vs 0.80, *P* < .001; senior radiologists: AUC = 0.79 vs 0.88, *P* < .001).

## Introduction

Lymph nodes (LNs) are the most common sites of cancer metastasis. For rectal cancer (RC), the status of LN involvement (N staging), reflected by the number and location of metastatic LNs, is not only an important prognostic factor but is also important for therapeutic decisions (1,2). Local excision or total mesorectal excision (TME) is suggested for early-stage T1–T2 RC without LN metastasis (LNM), while patients with more locally advanced disease or LNM are usually treated with neoadjuvant chemoradiotherapy, followed by TME (1,3). Moreover, the range of radiation therapy and TME is also determined by the number and location of metastatic LNs. Therefore, it is of paramount importance to accurately identify nodal involvement with high accuracy.

MRI is the preferred method for preoperative staging in RC (4). Subjective assessment of LN at MRI with respect to size and morphologic criteria is the current standard process for N staging (4). However, it remains challenging to detect LNM, with a wide range of accuracy (62%–85%) and poor sensitivity (38%–66%) (5–7). This is largely because of the overlap of characteristics between benign and metastatic LNs (8). Moreover, manual assessment is time-consuming and susceptible to the radiologist’s level of experience. Therefore, robust methods are urgently needed to improve preoperative N staging.

Several studies have adopted artificial intelligence (AI) to predict LN status in patients with RC (9–11). Notably, these methods extract features only from the primary tumor to indirectly predict LN status, inconsistent with the clinical imaging diagnosis workflow. A more logical diagnostic model is needed to directly assess the metastatic status of each LN. However, a major challenge lies in establishing the pathologic reference standard on a node-by-node basis for a large retrospective series to train and test an AI model (12). A previous AI model developed for directly assessing metastatic LN was based on the subjective assessment of radiologists rather than the pathologic reference standard (13).

Weakly supervised learning can be used to annotate each instance in a bag containing multiple instances when only the bag-level label is available (14,15). By considering a patient’s body as a collection (ie, bag) containing multiple LNs, we aimed to adopt weakly supervised learning to build an LN diagnosis model with MRI data and patient-level pathologic information. We also evaluated the model’s efficacy in assisting radiologists.

## Materials and Methods

Approval from the institutional review board of participating hospitals and a waiver for informed consent were obtained for this retrospective study.

### Study Design and Patients

**Figure 1:**
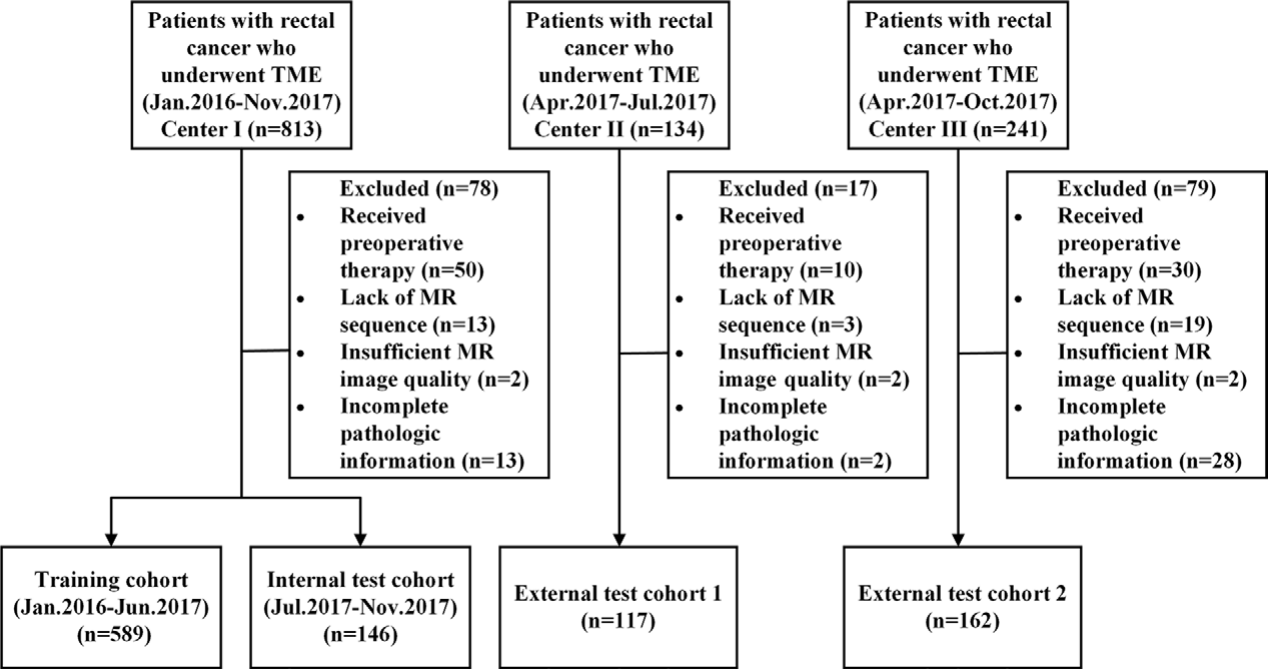
The flow diagram of patient enrollment.

In this retrospective multicenter diagnostic study, we collected data from three hospitals of consecutive patients with RC who underwent surgery (Fig 1). The inclusion criteria included the following: *(a)* pathology-confirmed diagnosis of rectal adenocarcinoma, *(b)* preoperative MRI scan available within 1 week prior to the initiation of TME surgery, and *(c)* availability of clinicopathologic data. The exclusion criteria included the following: *(a)* patient underwent preoperative therapy, *(b)* lack of MRI sequences, *(c)* insufficient MR image quality, and *(d)* incomplete pathologic information.

In total, 1014 patients with RC were recruited. A total of 735 patients from center 1 between January 2016 and November 2017 were divided into a training cohort (*n* = 589) and an internal test cohort (*n* = 146) at a ratio of 4:1 in chronological order. An external test cohort of 117 patients from April 2017 to July 2017 at center 2 was used. Furthermore, another external test cohort of 162 patients were from center 3 between April 2017 and October 2017.

**Figure 2:**
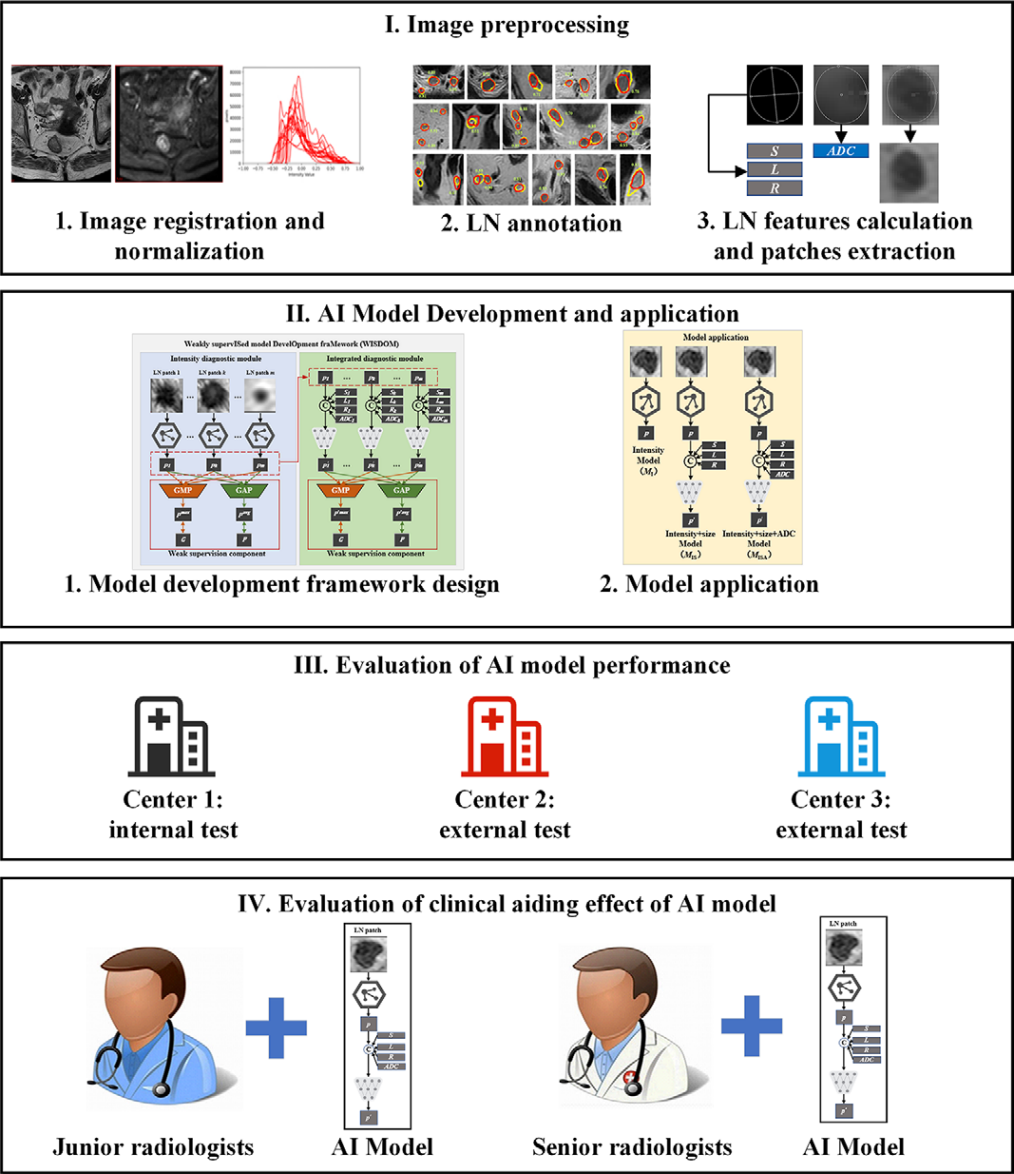
The schematic workflow of this study. The MR images were resampled and normalized, then the lymph nodes (LNs) were annotated, the features of each LN were calculated, and the LN patches were extracted. A model development framework was designed for using the LN features and patches to train diagnostic networks, and the networks were connected to form the LN diagnosis model. Model performance was evaluated with multicenter testing, and the effect of the built model in assisting radiologists was also evaluated.

The schematic workflow of this study is illustrated in Figure 2.

### Image Acquisition and Preprocessing

MRI sequences included axial T2-weighted imaging and diffusion-weighted imaging. Apparent diffusion coefficient (ADC) maps were generated (Appendix S1). Some imaging protocol differences across centers were addressed by image preprocessing (Appendix S2).

A fully automated LN detection and segmentation model, namely auto-LNDS, was used to annotate the LN masks (16). Then, the LN masks were revised by two radiologists (W.H. and Y.C., with 10 and 15 years of pelvic MRI experience, respectively), using Medical Imaging Interaction Toolkit software (MITK version 2016.11.0; *www.mitk.org*). For discrepancies in LN detection and segmentation between the two readers, an additional third senior radiologist (X.Y., with 30 years of experience) was employed for arbitration (Appendix S2 and Fig S1).

Based on the LN mask, the most common features used in LN assessment (17–19), including the long- and short-axis diameters, the ratio of short and long diameters, and the mean ADC value, were automatically calculated using OpenCV in Python (version 4.5.3.56). The LN patches were automatically extracted from T2-weighted imaging (Appendix S3).

### Development of LN Diagnosis Model

The diagram of the Weakly supervISed model DevelOpment fraMework (WISDOM) model is illustrated in Figure S3, which includes the intensity diagnostic module and the integrated diagnostic module.

This intensity diagnostic module (Appendix S4) was designed to train the ResNet-based (20) intensity diagnostic network to calculate the intensity-based metastatic probability. Inspired by weakly supervised learning (14,21,22), a weak supervision component was designed for matching the LN-level metastatic probability to the patient-level label, as follows: *(a)* the multiple-instance learning strategy implemented by the global maximum pooling layer: the LN with the highest metastatic probability *p*^max^ on an MR image would be metastatic (or nonmetastatic) if the patient had (or had no) postoperatively confirmed LNM; *(b)* the learning from label proportions strategy implemented by the global average pooling layer: the average metastatic probability *p*^avg^ of LNs on an MR image would be associated with the proportion of metastatic LNs in the resected LNs. Meanwhile, gradient-weighted class activation mapping (23) was used to generate heatmaps for identifying the metastasis-related region.

The integrated diagnostic module (Appendix S5), composed of a multilayer perceptron-based integrated diagnostic network and the same weak supervision component, was designed to combine the intensity-based metastatic probability, the size features, and the ADC value.

Three WISDOM models, including the intensity model *M*_I_, the intensity + size model *M*_IS_, and the intensity + size + ADC model *M*_ISA_, were developed to be compatible with different data inputs. Training procedures are described in Appendix S6. Our code is available on GitHub *(https://github.com/xiawei999000/WISDOM*).

### Evaluation of WISDOM Model for Aiding Radiologists in N Staging

To evaluate the aiding effect of the WISDOM model, junior and senior radiologists groups (<3 years and >10 years of experience, respectively), with three radiologists in each group, were invited to review all MR images in the three cohorts. All radiologists were masked to the clinical and histologic information. The interpretations consisted of two components. First, the initial review process and diagnosis were made by the two radiologist groups alone. The three radiologists in each group performed qualitative N staging according to recognized criteria (17) and reached majority or consensus. Next, readers in each group used the diagnoses and the heatmaps of the model to aid diagnosis 1 month later (Appendix S7). The readers’ diagnosis was compared with the diagnosis from the model. If the two did not match, readers could choose to adopt the model’s diagnosis or adhere to their own diagnosis as the final diagnosis. A comparison of each radiologist’s two assessments was also conducted.

### Statistical Analysis

Comparisons were performed using χ^2^ or Fisher exact test for categorical variables and the *t* test or Mann-Whitney *U* test for continuous variables, as appropriate. The performance of binary N staging (non-LNM or LNM) was evaluated by receiver operating characteristic (ROC) curve analysis. Comparisons of area under the ROC curve (AUC) values were assessed by DeLong test. A *P* value of .05 or less was considered statistically significant in two-tailed analyses. The clinical usefulness and calibration of models were evaluated with decision curve and calibration curve analysis, respectively.

The mean absolute error (MAE) was calculated to evaluate the differences between the identified metastatic LN number and the pathology-confirmed metastatic LN number. Furthermore, we calculated the concordance index (C index) for ternary N staging as N0 (no metastatic LNs), N1 (one to three metastatic LNs), and N2 (four or more metastatic LNs). Confusion matrices in ternary N staging are depicted, and the κ coefficient and F1 scores are also reported. The performances were evaluated in each test cohort, and the overall performances were calculated in an overall test cohort containing the patients of all test cohorts. Details are in Appendix S8. Statistical analysis was conducted with R (version 4.0.0, *https://www.r-project.org/*).

## Results

### Clinicopathologic Data

A total of 1014 patients (median age, 62 years; IQR, 54–68 years; 590 male, 424 female) were included. The clinicopathologic characteristics are summarized in the Table. There were significant differences in age, pathologic T stage, and size of LN across cohorts. There was no evidence of a difference in pathologic N stage across cohorts (*P* = .09), with LNM rates of 39.0% and 36.3% for the training cohort and internal test cohort and 34.2% and 45.0% for external test cohorts 1 and 2, respectively. A total of 14 686 LNs from the 1014 patients (mean, 14 ± five [SD] LNs per patient; range, five to 50) were harvested after surgery, among which 1637 LNs (1.6 ± 3.2 LNs per patient; range, 0–35) were metastatic.

**Table:**
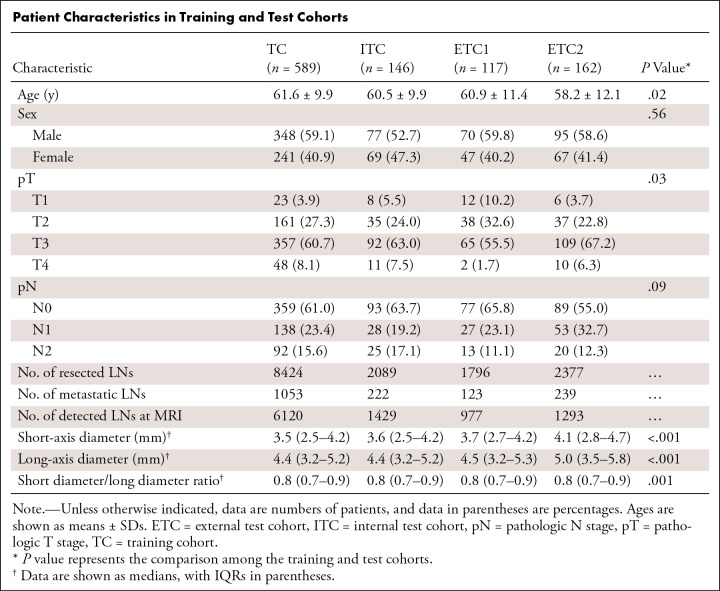
Patient Characteristics in Training and Test Cohorts

### LN Diagnosis Performance

For binary N staging (Fig S6), the *M*_I_ model achieved an overall AUC of 0.78 (95% CI: 0.73, 0.82). By integrating the intensity and size features, the overall AUC of *M*_IS_ increased to 0.80 (95% CI: 0.76, 0.84; *P* = .006). The *M*_ISA_ model, further introducing ADC values, yielded higher AUCs than *M*_IS_, with an overall AUC of 0.81 (95% CI: 0.77, 0.86; *P* = .04). The specificity of the above three AI models exceeded 80% in all cohorts. However, the overall sensitivity remained lower, albeit improving from 61.9% (95% CI: 54.1%, 69.3%) to 70.2% (95% CI: 62.7%, 77%). Additionally, the decision curve analysis (Fig S7) and calibration curves (Fig S8) graphically indicated that *M*_ISA_ had the largest net benefit and best calibration in the overall test cohort.

For the number of metastatic LNs (Fig S6), all models produced an MAE of about one LN. When adding the size features, the MAE decreased from 1.103 (95% CI: 0.925, 1.281) to 1.075 (95% CI: 0.896, 1.253), and the *M*_ISA_ model achieved the lowest MAE of all cohorts, with an overall MAE of 1.049 (95% CI: 0.875, 1.223).

For ternary N staging (Fig S9), *M*_I_ achieved an overall C index of 0.730 (95% CI: 0.692, 0.769). By integrating the intensity and size features, *M*_IS_ obtained a higher overall C index of 0.739 (95% CI: 0.700, 0.778; *P* = .02). The *M*_ISA_ model produced the best results among the above models, with an overall C index of 0.765 (95% CI: 0.727, 0.802; *P* = .03). The classification confusion matrices are presented in Figure S10. The accuracy of the model in identifying N0 was around 80%, while the accuracy of identifying N1 and N2 was relatively low.

### Interpretability of the WISDOM Model

**Figure 3:**
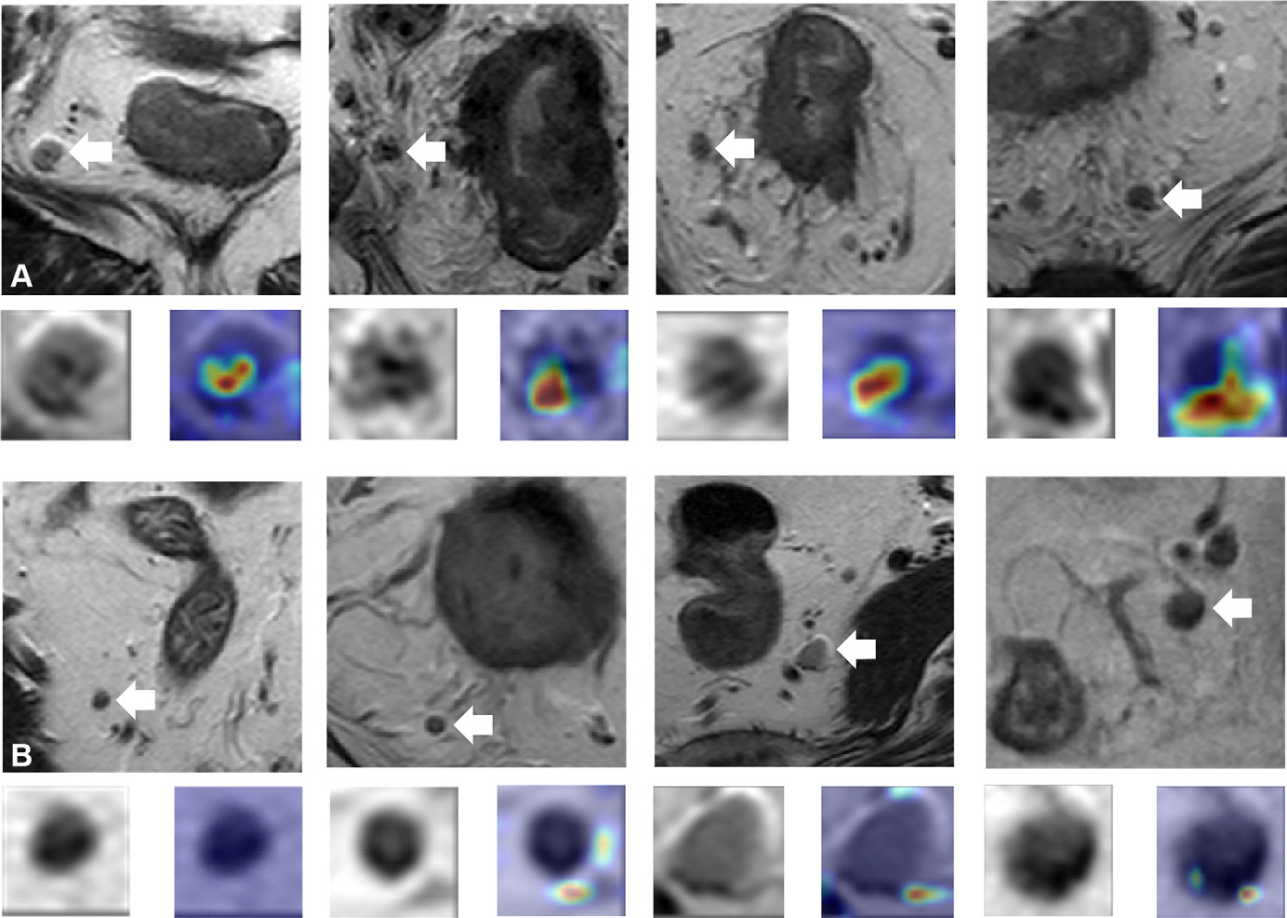
Examples of lymph node (LN) images. In each axial T2-weighted MR image, the white arrow points out an LN. The LN is magnified and displayed at the bottom left, and the attention heatmap generated by the diagnostic model is shown in the bottom right. The hot regions with deeper red correlate to higher relevance for LN metastasis. **(A)** LN images in patients with LN metastasis. **(B)** LN images in patients without LN metastasis.

To better comprehend the WISDOM model, heatmaps were generated to highlight the important regions. Two types of regions valuable for LN diagnosis on the MR images (namely, the intranodal and perinodal regions) were identified (Appendix S9). Figure 3 shows how the WISDOM model highlights regions it has predicted as positive, which might aid radiologists by quickly drawing their attention to suspicious areas. Moreover, the location of the hotspot derived from the model may vary, with the intranodal or perinodal region highlighted in 78.3% of model-defined LNM patches, compared with only 29.6% in model-defined non-LNM patches (Table S2). Furthermore, the short- and long-axis diameters, as well as the ADC values of the model-defined metastatic LNs, were significantly higher than those of the model-defined nonmetastatic LNs (all *P* < .001) (Appendix S10).

### The Effect of the WISDOM Model in Aiding Clinical Diagnosis

**Figure 4:**
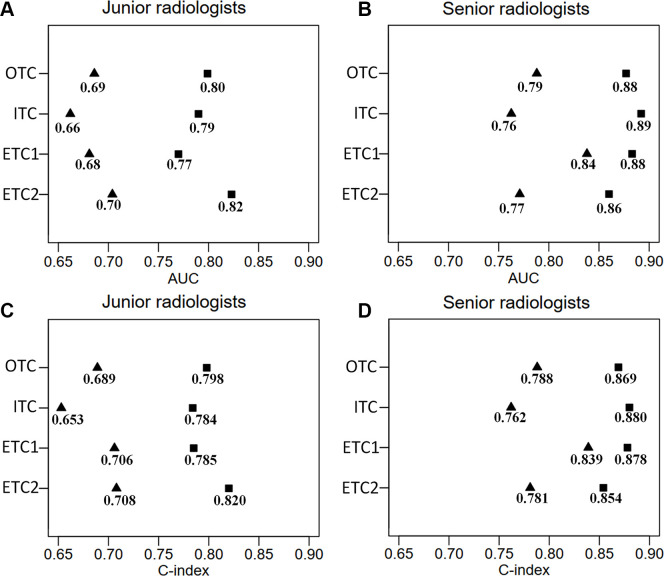
The area under the receiver operating characteristic curve (AUC) and concordance index (C index) of radiologists for each test set. Performance of radiologists without (triangle) and with (square) the assistance of the Weakly supervISed model DevelOpment fraMework (ie, WISDOM) are displayed. **(A)** AUC of junior radiologists. **(B)** AUC of senior radiologists. **(C)** C index of junior radiologists. **(D)** C index of senior radiologists. ETC = external test cohort, ITC = internal test cohort, OTC = overall test cohort.

**Figure 5:**
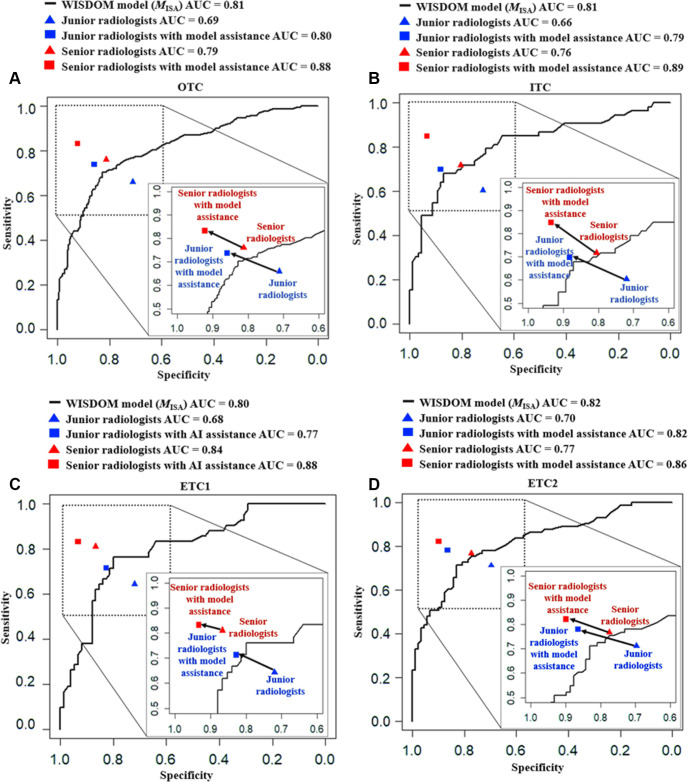
The receiver operating characteristic (ROC) curves of the Weakly supervISed model DevelOpment fraMework (WISDOM) model and radiologists (without and with model assistance) for each test set. **(A)** Overall test cohort. **(B)** Internal test cohort. **(C)** External test cohort 1. **(D)** External test cohort 2. AUC = area under the ROC curve, *M*_I_ = intensity model, *M*_IS_ = intensity + size model, *M*_ISA_ = intensity + size + apparent diffusion coefficient model.

To evaluate the WISDOM-aided diagnostic process in binary N staging, the performance of the best WISDOM model (*M*_ISA_) was compared with radiologists in all test cohorts. The overall AUC for the junior radiologists was only 0.69 (95% CI: 0.64, 0.73), which was significantly lower than that of the model (*P* < .001), and was augmented to 0.80 (95% CI: 0.76, 0.84) with model assistance (*P* < .001) (Fig 4 and Table S3). For the senior radiologists, performance was close to the model (0.79 vs 0.81, *P* = .21), and the overall AUC significantly improved to 0.88 (95% CI: 0.85, 0.91; *P* < .001) when assisted by the model. The ROC curves are plotted in Figure 5. Moreover, the sensitivity and specificity also improved after the aid of the model, especially for the junior radiologists (66.0% vs 73.8%, *P* = .06 and 71.2% vs 85.9%, *P* < .001, respectively). The MAE was also further reduced with model assistance for the radiologists.

For ternary N staging, similar trends were observed (Fig 4 and Table S4). The C index for radiologists alone ranged from 0.653 (95% CI: 0.581, 0.725) to 0.839 (95% CI: 0.780, 0.898), and their performance was significantly improved when assisted by the model, for both senior (*P* = .007) and junior (*P* < .001) radiologists. Overall, the diagnostic accuracies, κ coefficient, and F1 scores of radiologists also improved.

The overall AUC and C index of each radiologist was also calculated (Tables S5–S7). For all cases in the overall test cohort or for the cases where the radiologists were not in complete agreement before using the model, it was shown that the model could significantly improve the performance of each radiologist. For the cases where the model agreed with most radiologists, the model could significantly improve the performance of one junior radiologist and two senior radiologists.

## Discussion

In this multicenter study, an LN diagnosis model was developed for RC by using a weakly supervised learning framework, WISDOM, using MRI data and the patient-level pathologic report. The WISDOM model had favorable test performance in three cohorts (overall AUC = 0.81). More importantly, the performance of all radiologists was improved with model assistance (junior radiologists: AUC = 0.69 vs 0.80, *P* < .001; senior radiologists: AUC = 0.79 vs 0.88, *P* < .001).

Previous studies have indirectly assessed LNM by extracting radiomics features of primary rectal tumors (12). Meng et al (9) and Yang et al (24) developed radiomics models with AUC values of 0.677 and 0.770, respectively. Liu et al (25) built a multiregional radiomics model based on the primary tumor and mesorectum (AUC = 0.702). Similarly, a radiomics model of the most visible LNs on T2-weighted images investigated by Li et al (26) also showed good performance (AUC = 0.92). However, these radiomics findings are all of limited clinical translation potentially because of the small sample size and the lack of multicenter testing and, most importantly, none of these studies identified the number and location of metastatic LNs, which is inconsistent with the clinical diagnosis workflow. More recently, one study used a Faster R-CNN model to automatically detect metastatic LNs (AUC = 0.92), but the reference standard of labeling LNs was based on the subjective recognition of radiologists (13). Moreover, the MRI protocol utilized 6-mm thick, 1.5-mm spacing, and without a small field of view, which is not in compliance with recognized standards and may preclude the detection of small LNs. As such, the analysis was mainly based on large LNs, which may overestimate performance.

It is not feasible to individually match LNs detected at MRI with the pathologic node in large retrospective studies, especially for nodes smaller than 3 mm. The WISDOM model could mine the associations between the routine MRI data and the patient-level pathologic information and identify the location and number of suspicious metastatic LNs, which is consistent with the diagnosis workflow of radiologists. Of note, the WISDOM model is tested across centers, and the robust performances further confirmed the generalizability. Furthermore, the WISDOM model could be combined with the previously developed auto-LNDS model, which fully automated the detection and segmentation of LNs in less than 2 seconds, providing an efficient preoperative N staging method.

The superior performance of WISDOM was probably due in part to the integration of different LN characteristics. The T2-weighted image intensity characteristics of LNs could be automatically analyzed by WISDOM. The performance of the model was further improved by introducing LN size features and ADC values. The significant difference between the model-defined metastatic and nonmetastatic LNs in terms of short- and long-axis diameters, as well as the ADC values, was consistent with a previous study (19), which further enhanced the reliability of WISDOM. More importantly, the model could highlight the metastasis-relevant regions. As demonstrated by the heatmaps, most of the hotspot regions were located within nodal or perinodal regions, which might have heterogeneous intensity or irregular morphology to the radiologists, indicating that the model could decode subtle image characteristics, further enhancing the interpretability and rationality of the model, which was also useful in aiding clinicians by rapidly drawing attention to metastasis-related characteristics.

Real-world clinical decisions should be supervised by clinicians even if AI is reported to have superior performance. To our knowledge, this is the first attempt to evaluate an AI tool aiding MRI-based LN diagnosis in RC. Intriguingly, auxiliary use of the model showed advantages in improving accuracy, especially for junior radiologists, who were more inclined to accept the recommendations of the model than were senior radiologists, which may be related to a relative lack of confidence and work experience. Notably, for some patients with pathology-confirmed LNM, the model successfully identified some small but suspicious metastatic LNs that were neglected by radiologists. Meanwhile, for some patients with pathology-confirmed negative LNs, the model also correctly diagnosed some LNs with large size or other concerning imaging characteristics misdiagnosed by radiologists. Thus, the model could help radiologists to reduce both understaging and overstaging errors.

There were some limitations in this study. First, limited by the retrospective nature of this study, the pathology reference standard of each individual LN detected at MRI was not available. Future research based on precise node-by-node matching of histologic whole-mount specimens and the MR images will be conducted to further evaluate the model at the LN level. Second, due to the limited resolution of the MR images, some small metastatic LNs may not have been identified, which may have decreased model performance and manifested with lower sensitivity. Third, the proximity between suspicious LNs and the mesorectal fascia is important to report for surgical planning. However, this study focused on the LN itself and did not analyze the proximity. Fourth, rare types of tumors such as mucinous adenocarcinoma and signet ring cell carcinoma were not included in this study, and the model was not trained to classify mucinous LNs. Finally, this study included only patients who underwent TME directly and annotated the LNs within the mesorectum; thus, the model was not trained for the evaluation of nonregional LNs and posttreatment follow-up.

In conclusion, we developed WISDOM, an AI model for preoperative LN diagnosis at MRI in patients with RC. The performance and generalizability of the WISDOM model and the improved radiologist performance highlight the potential for application in the accurate staging and individualized treatment planning for patients with RC.
